# Development and Treatment of Albuminuria

**Published:** 1868-02

**Authors:** 


### DEVELOPMENT AND TREATMENT OF ALBUMINURIA.

*To the Editors of the Boston Medical & Surgical Journal:*

In view of the notorious inefficacy of our treatment of Bright's disease, I thought the following note might be interesting. It is a condensation of an article read before the French Academie Impériale de Médecine, by Prof. Semola of Naples, and is the complement of a previous memoir by the same author. In his previous communication, Prof. Semola developed the opinion that the passage of the albumen into the urine, in Bright's disease, was necessary consequence of a general deficiency of nutrition, by which the albumen, rendered incapable of performing its functions, was eliminated by the kidneys as a substance foreign to the organism. According to this theory, the alterations of these organs would play only a secondary rôle in the development of the disease; and although the condition of the kidneys is of value in prognosis, Prof. Semola protests against the ideas of those who pretend to explain or resolve the question by purely anatomical deductions.

One diagnostic sign distinguishing organic from symptomatic albuminuria is furnished by the quantity of the urea, which in true Bright's disease diminishes with the first appearance of the albumen, and, at a later period, accumulates in the blood. The same is true of the sulphates. Of the artificial albuminurias, that produced by the suppression of the cutaneous functions is the most like Bright's disease. This suppression both prevents the oxidation of the materials introduced into the system in the form of peptones (the products of the digestion of albumen), and causes a congestion of the viscera and especially of the kidneys.

Thus, according to this author, Bright's disease is not the result of a primitive anatomical lesion of the kidneys, but is a result of this double series of effects which succeed the more or less sudden suppression of the functions of the skin.

The aim of the physician should be to reëstablish these functions, and thus increase the oxidation of the peptones, and relieve the renal congestion.

Among the means best suited to this purpose are the ordinary sweatings, or, in obstinate cases, hot air baths always followed by cool or cold shower baths; preparations of arsenic, and inhalations of oxygen. The diet should be vegetable or starchy, with but very little meat.

[*Boston Med. and Surg. Journal.*] W. F. M.

